# High self-paternity levels and effects of fertilised-seed number on size of strawberry fruit

**DOI:** 10.1371/journal.pone.0273457

**Published:** 2022-09-13

**Authors:** Wiebke Kämper, Cao Dinh Dung, Steven M. Ogbourne, Helen M. Wallace, Stephen J. Trueman

**Affiliations:** 1 Centre for Planetary Health and Food Security, School of Environment and Science, Griffith University, Nathan, Australia; 2 Centre for Bioinnovation, University of the Sunshine Coast, Sippy Downs, Australia; 3 School of Science, Technology and Engineering, University of the Sunshine Coast, Sippy Downs, Australia; Institute of Mediterranean Forest Ecosystems of Athens, GREECE

## Abstract

Cross-pollination can increase fruit production in both self-incompatible and self-compatible fruit crops. However, it is often unclear what proportions of the fruit crop result from cross-pollination. We quantified the proportion of cross-pollinated seeds and the proportion of fertilised seeds in two strawberry cultivars, Red Rhapsody and Sundrench, at increasing distances from a cross-pollen source. We assessed whether fully self-pollinated fruit and partly cross-pollinated fruit differed in fruit size, colour, firmness, Brix and acidity. We also assessed whether fruit size and quality were affected by the number or percentage of fertilised seeds. Almost all seeds of both cultivars resulted from self-pollination (~98%), even at only 1 m from a cross-pollen source. Distance from a cross-pollen source did not affect the proportion of partly cross-pollinated fruit or the proportion of cross-pollinated seeds per fruit. The mass and diameter of fully self-pollinated Sundrench fruit, and the redness and Brix of fully self-pollinated Red Rhapsody fruit, were higher than partly cross-pollinated fruit. Fruit mass, length and diameter increased, and acidity decreased, with increasing numbers of fertilised seeds in both cultivars. Fruit mass also increased with the percentage of fertilised seeds. Our results show that cross-pollination was not required for Red Rhapsody and Sundrench fruit production, and that cross-pollination was a rare occurrence even close to cross pollen source. Self-pollen deposition on stigmas is required to maximise the number of fertilised seeds, and consequently fruit size and quality. Our research indicates that bees improve strawberry fruit size by increasing the number of stigmas that receive pollen. Our results suggest that placing bee hives on strawberry farms during flowering and establishing nearby pollinator habitat to support wild pollinators could improve strawberry yield and fruit quality.

## Introduction

Plant reproduction often depends on animal pollination, and many crops depend on pollination for optimal crop production and crop quality [1–4]. The genotype of pollen deposited on the stigmas can also affect crop yield and quality. For example, cross-pollination is essential for the successful reproduction of self-incompatible crops, and cross-pollination can improve the fruit or seed set, size, and quality of many self-compatible crops [3–9]. Cross-pollination in clonally-propagated crops occurs when pollen from one cultivar is transferred to the stigma of another cultivar. Self-pollination occurs when pollen of one cultivar is transferred to the stigma of the same flower, to the stigma of another flower within the same plant, or even to the stigma of another plant of the same cultivar. Many crops are planted in single-cultivar blocks, and so cross-pollen often needs to be transferred long distances by pollinators such as bees [4, 10, 11].

The flowers of many berry crops such as strawberry (*Fragaria × ananassa* Duch.) contain hundreds of free simple carpels [12]. The carpels are embedded within the receptacle, which enlarges after the ovaries expand following successful pollination and fertilisation [12]. The resulting reproductive structure is an aggregate fruit, with the fleshy part being derived from the receptacle that holds the ovaries, rather than from the ovaries themselves [13]. Strawberry flowers are generally self-compatible [14, 15]. The number of fertilised ovules determines fruit mass, length, diameter and firmness, whereas the pollen donor affects fruit colour and Brix, when all stigmas on a self-compatible Redlands Joy strawberry flower are pollinated by the same pollen source under glasshouse conditions [15]. Insect pollinators are known to improve strawberry fruit quality since bee-pollinated and open-pollinated flowers on strawberry farms produce heavier and sweeter fruit with longer shelf life than flowers that are bagged to exclude pollinators [8, 14, 16]. It is unclear whether these differences between autogamous pollination and open-pollination were caused by a greater number of stigmas receiving pollen (of any genotype) or by bee-pollinated and open-pollinated flowers receiving more cross-pollen. These differences are further complicated by the fact that individual strawberry fruit may carry both self-pollinated and cross-pollinated seeds, and it is unclear how different levels of cross-pollination within an individual fruit affect fruit size and quality.

Here, we aimed to understand the contribution of self-pollination and cross-pollination to strawberry yield and quality. In particular, we aimed to determine whether (i) the proportion of partly cross-pollinated fruit and (ii) the proportion of cross-pollinated seeds per fruit varied at different distances from a cross-pollen source in an open-pollination farm setting. Moreover, we aimed to determine whether (iii) pollen parentage affected fruit size, colour, firmness, seed filling, Brix or acidity, and whether (iv) the percentage or (v) the number of fertilised seeds affected fruit size and quality. We hypothesised that there would be a greater proportion of fruit with cross-pollinated seeds close to a cross-pollen source, and that fruit size and quality would be affected by pollen paternity and by the percentage and number of fertilised seeds.

## Material and methods

### Study sites and design

**Fig 1:**
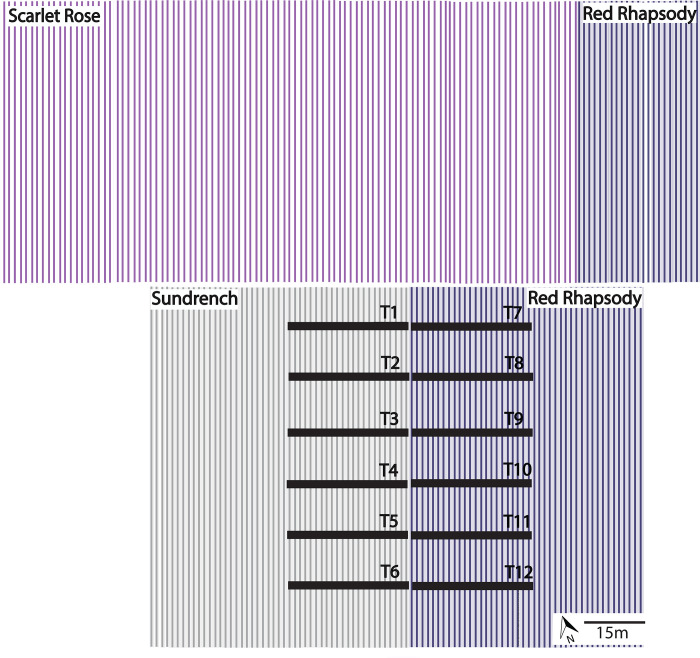
Strawberry farm design, consisting of 49 rows of cultivar Sundrench next to 44 rows of cultivar Red Rhapsody. ‘T1’ to ‘T6’ show the six sampling transects for cultivar Sundrench, and ‘T7’ to ‘T12’ show the six sampling transects for cultivar Red Rhapsody. North of the experimental block are 103 rows of cultivar Scarlet Rose next to another 19 rows of Red Rhapsody. Flower visitors were observed and fruit collected from each transect at rows 1, 3, 10 and 20 from the other cultivar.

We conducted the study on a commercial strawberry farm (26°45’27”S 153°03’30”E) in southeast Queensland, Australia. The farm contained three self-compatible strawberry cultivars, Red Rhapsody, Sundrench and Scarlet Rose. We undertook the study in a block that comprised 44 consecutive rows of a single cultivar, Red Rhapsody, and 49 consecutive rows of another cultivar, Sundrench (Fig 1). The neighbouring block on one side consisted of 103 rows of Scarlet Rose and 19 rows of Red Rhapsody. The neighbouring block on the other side, and the remainder of the farm, consisted of cultivar Red Rhapsody, except for one distant block of Scarlet Rose.

**Fig 2:**
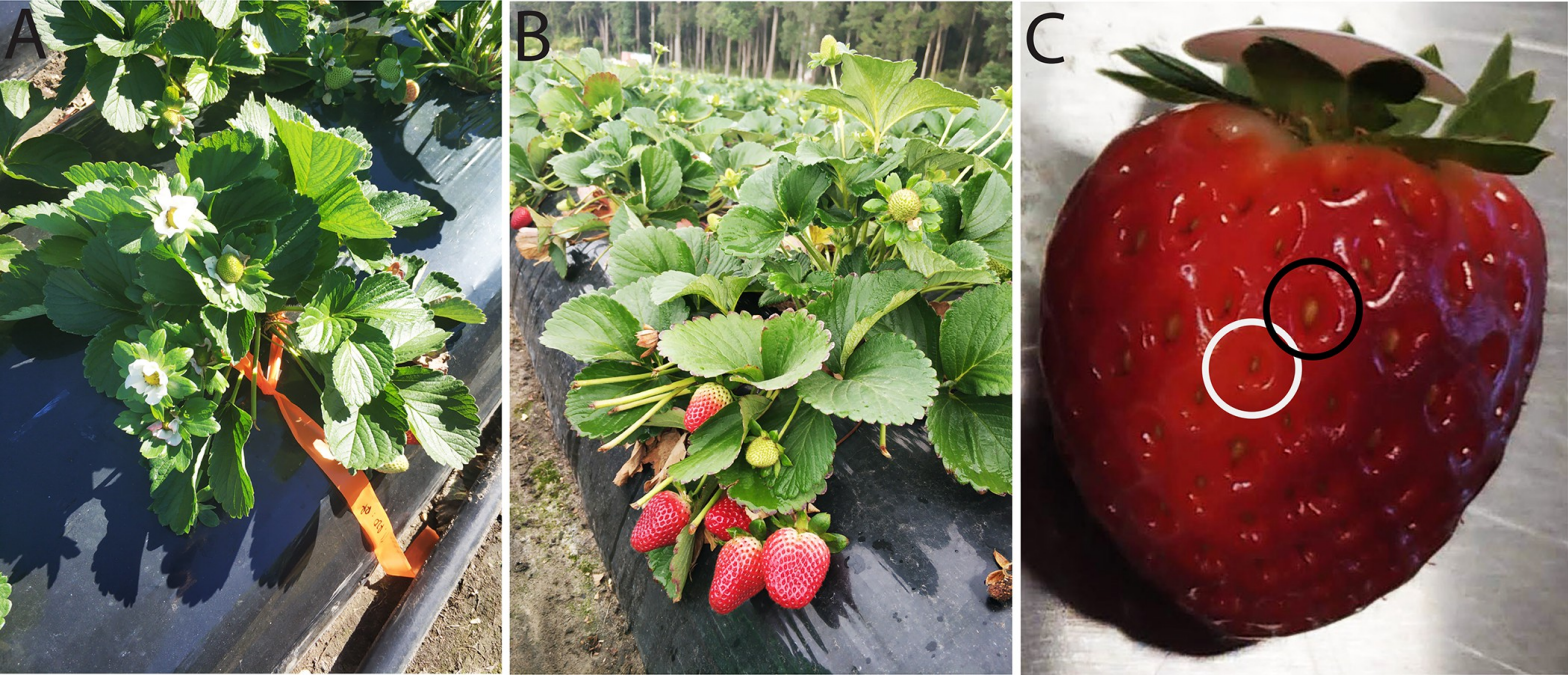
Red Rhapsody and Sundrench strawberry plants (A) during flowering and (B) at harvest, and (C) a Red Rhapsody fruit with an unfertilised seed (white circle) and a fertilised seed (black circle).

A total of 24 study plants for each cultivar, Red Rhapsody and Sundrench, was located along six transects. Each transect consisted of a study plant in the first row (1 m), third row (3 m), tenth row (10 m) and twentieth row (20 m) from the other cultivar. We spaced the transects approximately 2 m apart along the rows. Twenty honeybee hives were located about 100 m from the study plants. We counted the numbers of (a) honeybees, (b) stingless bees, (c) syrphid flies, (d) other insects, and (e) other animals that contacted a flower within a 5-min period between 1000 hours and 1600 hours in a 1 m^2^ quadrat around each experimental plant. Flower visitors were counted on four cloudless days of the flowering season: (a) 24 and 28 June 2019, and (b) 29 July and 1 August 2019 (Fig 2A).

We harvested fruit of each cultivar at commercial maturity, i.e. when the skin was becoming fully red (Fig 2B). Harvesting occurred on 25 July and 28 August 2019 because it takes approximately 4 weeks from flower anthesis for the fruit to reach commercial maturity. Three mature fruit were sampled on each occasion from each study plant or, if this plant did not carry three mature fruit, the remaining fruit were sampled from the closest available plant. We sampled a total of 143 Red Rhapsody and 144 Sundrench fruit.

### Sample processing

We recorded the fresh mass, length and diameter of each fruit, excluding the pedicel and sepals. We assessed the colour of each fruit using a CR-10 colorimeter (Konica Minolta, Chiyoda, Japan), which provided values for brightness, L*, where brighter is more positive and darker more negative, redness, a*, where redder is more positive and greener more negative, and yellowness, b*, where yellower is more positive and bluer more negative. We determined fruit firmness using an FR-5120 fruit hardness tester with a 3-mm head (Lutron Electronic, Taipei, Taiwan) by recording the maximum force needed to push the probe 6 mm vertically downwards into the fruit apex at a speed of 100 mm min^–1^ [17, 18]. The total numbers of seeds (i.e. achenes) and filled seeds (i.e. fertilised achenes) were counted on each fruit. Unfertilised seeds were visibly distinguishable from fertilised seeds due to their smaller size and abnormal morphology (Fig 2C). We then calculated the percentage of seeds that was fertilised on each fruit. Ten filled seeds from each fruit were selected in a stratified design for genotyping, with five seeds taken at uniform distances along each of two lines from the top to the bottom of the fruit. Fruit were then frozen at -20°C prior to other quality analyses. Fruit were individually defrosted for 90 s (1000 W, 2450 MHz), squeezed, and the released juices were filtered through filter paper. We determined total soluble solid concentration (TSS; i.e. °Brix) and acid concentration of the filtered extract using a PAL-BX|ACID4 sugar and acidity meter (Atago, Tokyo, Japan). Brix:acid ratio was then calculated for each fruit.

### Paternity testing

DNA extraction followed the protocol for glass-fibre plate DNA extraction for plants (http://ccdb.ca/resources/) [21]. We added disposable 2.3 mm and 0.1 mm zirconia/silica beads prior to shaking on a TissueLyser II (Qiagen, Hilden, Germany) [22]. The DNA of each sample was amplified at three microsatellite loci that distinguish the three cultivars at our study site [19, 20]. No other strawberry farm was located within the flight radius of honeybees (~3.5 km). The 5′ end of each forward primer was fluorescently labelled (Table 1). One multiplex PCR was performed per sample using a Qiagen Type-it Microsatellite PCR Kit. Reactions were performed in 12.5 μL volumes containing approximately 20 ng DNA template, 5.6 μL Type-it Multiplex PCR Master Mix, 2 μM of each primer and 3.6 μL RNase-free water. PCR was performed with initial denaturation at 95°C for 5 min, followed by 32 cycles of 95°C for 30 s, 57°C for 90 s, and 72°C for 30 s, followed by final elongation at 60°C for 30 min.

**Table 1:** Characterisation of three polymorphic microsatellite loci used to determine paternity of strawberry seeds [19, 20].

Locus	Primer sequences (5’ to 3’)	Repeat motif	Fluorescent label	Size range	Accession number
EMFax380097	GTTTTGCTTGGAGGTGTAAAGG	(CT)_7_	NED, VIC	155–200	CO380097
	GCTGCTGCTCTCTTGTAATGTG				
CFACT084	AAAACTAGGCGGTGTTGCAG	(GA)_9_	PET, FAM	102–143	AM691781
	GAACAGATCCACCGAGCAGT				
EMFn125	CCCAACCCTAAACCATACCC	(CT)_8_	PET, FAM	200–222	AM051329
	ATGGTTGCCTTTGATTCACG				

We generated genotypes using an AB 3500 Genetic Analyser (Applied Biosystems, Foster City, CA) and scored allele sizes relative to an internal standard (600 LIZ® Size Standard, Applied Biosystems, Foster City, CA) using the program GeneMarker version 2.6.3 (SoftGenetics, State College, PA). Scoring was performed manually by scanning for alleles that uniquely identified one cultivar. A seed was considered cross-pollinated if we found an allele that occurs in the cross-pollen source (i.e. the paternal cultivar) but does not occur in the maternal cultivar. We then calculated the proportions of cross-pollinated seeds on each fruit.

### Statistical analysis

We used generalised linear models (GLMs) with binomial distribution and logit link function to test whether distance from a cross-pollen source affected the proportion of cross-pollinated seeds. We used linear mixed models with transect and harvest as random effects to test whether fruit on which all tested seeds were self-pollinated (hereafter termed ‘self-pollinated fruit’) and fruit on which at least one out of 10 seeds was cross-pollinated (hereafter termed ‘partly cross-pollinated fruit’) had different size or nutritional quality. Linear regressions were performed to analyse whether the number of fertilised seeds or the percentage of fertilised seeds per fruit affected fruit size or quality. Statistical analyses were performed using R version 3.6.2 [23]. Mixed models were performed with the packages, ‘lme4’ and ‘multcomp’ [24, 25].

## Results

### Flower visitors

Honeybees, stingless bees and other insects were the most abundant visitors to strawberry flowers at the study site (Table 2).

**Table 2:** Mean (± SE) number of flower visitors to strawberry flowers in a 1 m^2^ quadrat during a 5-min period on each of four days during peak flowering.

Flower visitor	Red Rhapsody	Sundrench
Honeybees	2.79 ± 0.36	3.38 ± 0.41
Stingless bees	1.58 ± 0.32	2.21 ± 0.36
Syrphid flies	0.38 ± 0.17	0.17 ± 0.08
Other insects	1.96 ± 0.33	1.54 ± 0.28
Other animals	0.08 ± 0.06	0.13 ± 0.07

### Levels of cross-paternity

**Fig 3:**
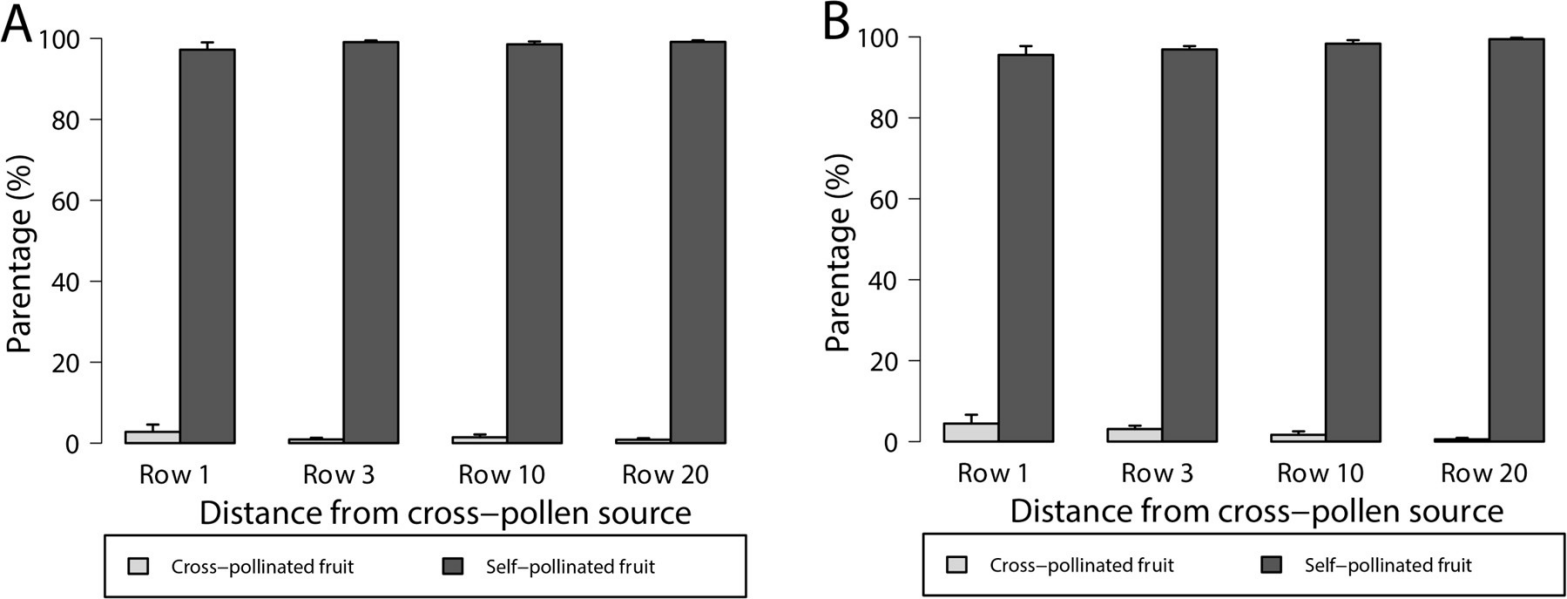
Percentage of partly cross-pollinated and fully self-pollinated fruit of (A) Red Rhapsody and (B) Sundrench strawberry at different numbers of rows from a cross-pollen source. Fruit were sampled along transects starting at plants adjacent to another cultivar (Row 1) and ending in the middle of a single-cultivar block (Row 20). Means (+SE) for cross-pollination levels for each cultivar are not significantly different across rows (GLM, p > 0.05, n = 6).

Only 1–3% of Red Rhapsody fruit and 1–4% of Sundrench fruit were partly cross-pollinated, while all remaining fruit were fully self-pollinated (Fig 3). The proportions of fruit that were partly cross-pollinated did not differ significantly with distance from a cross-pollen source (Fig 3; Red Rhapsody: *Z* = -0.19, *P* = 0.85; Sundrench: *Z* = -0.41, *P* = 0.68). A total of 21 Red Rhapsody seeds was cross-pollinated, with 16 seeds pollinated by Sundrench and five seeds identified as being pollinated by either Sundrench or Scarlet Rose. These cross-pollinated Red Rhapsody seeds were detected in 14 out of 143 fruit, four of which were collected at each of 1 row and 10 rows from a cross-pollen source and three of which were collected at each of 3 rows and 20 rows from a cross-pollen source. Eleven of the 14 partly cross-pollinated Red Rhapsody fruit had one cross-pollinated seed, and one fruit each had two, three or five cross-pollinated seeds. A total of 32 Sundrench seeds was cross-pollinated, with 30 seeds pollinated by Red Rhapsody and the remaining two seeds identified as being pollinated by either Red Rhapsody or Scarlet Rose. These cross-pollinated Sundrench seeds were detected in 28 out of 144 Sundrench fruit, with 12, ten, four and two fruit detected with cross-pollinated seeds at 1, 3, 10 and 20 rows from a cross-pollen source, respectively. Twenty-two of the 28 partly cross-pollinated fruit had one cross-pollinated seed, five fruit had two cross-pollinated seeds, and one fruit had three cross-pollinated seeds. The proportion of cross-pollinated seeds per fruit was not affected by distance from a cross-pollen source, i.e. number of rows, in either cultivar (Red Rhapsody: *Z* = 0.14, *P* = 0.89; Sundrench: *Z* = -0.13, *P* = 0.90).

### Paternity effects on fruit size and quality

Length, diameter and mass did not differ significantly between partly cross-pollinated and fully self-pollinated Red Rhapsody fruit (Table 3). Redness of partly cross-pollinated Red Rhapsody fruit was lower than redness of fully self-pollinated fruit, while brightness and yellowness did not differ significantly. Firmness, number of filled seeds, and percentage of filled seeds did not differ significantly between partly cross-pollinated and fully self-pollinated Red Rhapsody fruit. Brix was lower in partly cross-pollinated fruit than fully self-pollinated Red Rhapsody fruit, whereas acidity and Brix:acid ratio did not differ significantly.

**Table 3:** Size and quality of partly cross-pollinated and fully self-pollinated fruit of Red Rhapsody strawberry.

Fruit size and quality	Paternity		P
	Partly cross-pollinated	Fully self-pollinated	
Length (cm)	4.19 ± 0.05	4.36 ± 0.28	
Diameter (cm)	3.58 ± 0.04	3.58 ± 0.04	
Mass (g)	24.03 ± 0.51	24.10 ± 0.66	
Brightness (L*)	28.6 ± 0. 3	29.3 ± 0.3	
Redness (a*)	39.4 ± 0.3a	40.8 ± 0.3b	*
Yellowness (b*)	17.9 ± 0.4	19.7 ± 0.4	
Firmness (N)	2.84 ± 0.08	3.42 ± 0.09	
Filled seeds (n)	266 ± 5	270 ± 7	
Filled seeds (%)	94.3 ± 0.4	95.8 ± 0.4	
TSS^1^ (Brix)	5.99 ± 0.15a	6.91 ± 0.11b	*
Acidity (mg/g)	0.75 ± 0.02	1.65 ± 0.80	
Brix:acid	8.39 ± 0.30	8.82 ± 0.19	

Means ± SE with different letters are significantly different (mixed model

*P ≤ 0.05; n = 14–129)

^1^ Total soluble solid concentration

Diameter and mass of partly cross-pollinated Sundrench fruit were lower than fully self-pollinated fruit, while length did not differ significantly (Table 4). The colour of partly cross-pollinated and fully self-pollinated Sundrench fruit was similar. Fruit firmness, the number of filled seeds, and the percentage of filled seeds did not differ significantly between partly cross-pollinated and fully self-pollinated Sundrench fruit. Brix, acidity and Brix:acid ratio also did not differ significantly between partly cross-pollinated and fully self-pollinated Sundrench fruit.

**Table 4:** Size and quality of partly cross-pollinated and fully self-pollinated fruit of Sundrench strawberry.

Fruit size and quality	Paternity		P
	Partly cross-pollinated	Fully self-pollinated	
Length (cm)	3.80 ± 0.06	4.24 ± 0.08	
Diameter (cm)	3.31 ± 0.05a	3.63 ± 0.05b	*
Mass (g)	19.92 ± 0.73a	26.03 ± 0.95b	*
Brightness (L*)	30.6 ± 0.2	30.8 ± 0.3	
Redness (a*)	43.2 ± 0.4	44.8 ± 0.4	
Yellowness (b*)	18.3 ± 0.4	20.6 ± 0.4	
Firmness (N)	3.35 ± 0.09	3.73 ± 0.23	
Filled seeds (n)	215 ± 10	274 ± 11	
Filled seeds (%)	73.8 ± 2.1	79.3 ± 2.1	
TSS^1^ (Brix)	7.34 ± 0.15	6.57 ± 0.12	
Acidity (mg/g)	0.81 ± 0.02	0.67 ± 0.02	
Brix:acid	9.41 ± 0.17	10.78 ± 0.36	

Means ± SE with different letters are significantly different (mixed model

*P ≤ 0.05; n = 28–116)

^1^ Total soluble solid concentration

### Effect of fertilised seeds on fruit size and quality

Fruit length, diameter and mass increased with increasing numbers of fertilised seeds in both cultivars (Tables 5 and 6). Fruit length, diameter and mass also increased with increasing percentages of fertilised seeds in Sundrench (Table 6), but only fruit mass increased with increasing percentages of fertilised seeds in Red Rhapsody (Table 5).

**Table 5:** Coefficients of determination for linear regressions between the number or percentage of fertilised seeds and fruit size and quality parameters of Red Rhapsody strawberry fruit.

Fruit size and quality	Number of fertilised seeds		Percentage of seeds fertilised	
	r^2^	P	r^2^	P
Length (cm)	0.040	*	<0.001	
Diameter (cm)	0.33	***	0.02	
Mass (g)	0.42	***	0.03	*
Brightness (L*)	0.01		0.02	
Redness (a*)	0.005		0.006	
Yellowness (b*)	0.001		0.015	
Firmness (N)	0.007		0.001	
TSS (Brix)	0.019		<0.001	
Acidity (mg/g)	0.031	*	<0.001	
Brix:acid	0.025		0.014	

Significant linear regressions are indicated by asterisks

(* P < 0.05

*** P < 0.001; n = 14–129). All r values were positive.

**Table 6:** Coefficients of determination for linear regressions between the number or percentage of fertilised seeds and fruit size and quality parameters of Sundrench strawberry fruit.

Fruit size and quality	Number of fertilised seeds		Percentage of seeds fertilised	
	r^2^	P	r^2^	P
Length (cm)	0.69	***	0.32	***
Diameter (cm)	0.65	***	0.31	***
Mass (g)	0.68	***	0.26	***
Brightness (L*)	0.06		0.02	
Redness (a*)	0.001		0.003	
Yellowness (b*)	0.001		0.012	
Firmness (N)	0.003		0.006	
TSS (Brix)	0.14	***	0.15	***
Acidity (mg/g)	0.16	***	0.16	***
Brix:acid	0.004		0.007	

Significant linear regressions are indicated by asterisks

(*** P < 0.001; n = 28–116). The r values were positive, except for TSS and acidity.

Acidity, but not Brix or the Brix:acid ratio, of Red Rhapsody fruit increased with the number of fertilised seeds (Table 5). None of these parameters was affected significantly by the percentage of filled seeds on Red Rhapsody fruit (Table 5). Brix and acidity of Sundrench fruit both decreased with an increasing number or percentage of fertilised seeds and, as a result, Brix:acid ratio was not affected by the number or percentage of fertilised seeds (Table 6). Fruit colour and firmness were not affected by the number or percentage of fertilised seed in either cultivar (Tables 5 and 6).

## Discussion

Very few strawberry seeds (1–4%) resulted from cross-pollination in an open-pollination farm setting, regardless of proximity to a cross-pollen cultivar. Transfer of pollen from another cultivar was not essential for fruit production as almost all seeds of these self-compatible strawberry cultivars in an open-pollinated field were the result of self-pollination. We had hypothesised that there would be greater proportions of partly cross-pollinated fruit, and cross-pollinated seeds per fruit, close to a cross-pollen source. This was not the case, showing that strawberry production resulted almost entirely from self-pollination, even at the borders between two cultivars.

Each strawberry stigma in this open-pollinated field study had the potential to receive pollen from the same cultivar (i.e. self-pollen) or from two different cross-pollen sources. Each strawberry fruit could, thus, be the result of varying levels of cross-pollination and self-pollination, with seeds within one fruit possibly being the result of pollination by different pollen parents. However, we found that strawberry fruit were predominantly self-pollinated. No fruit had the majority of their seeds cross-pollinated, and so it was not possible to compare the size and quality of predominantly cross-pollinated fruit with the size and quality of predominantly self-pollinated fruit. However, we obtained some evidence that even low levels of cross-pollination might reduce fruit size and quality. Partly cross-pollinated Sundrench fruit had lower mass and diameter than fully self-pollinated fruit, and partly cross-pollinated Red Rhapsody fruit were less red and had lower Brix than fully self-pollinated fruit. The effect of different pollen parents on fruit characteristics is a phenomenon termed xenia [26, 27]. The cultivars in our study differ in their typical fruit characteristics, with Red Rhapsody producing large to extra-large firm fruit with a glossy dark-red appearance, Sundrench producing large soft fruit that can turn dark, and Scarlet Rose producing conical fruit with a bright glossy-red appearance [28–31]. Most partly cross-pollinated Red Rhapsody seeds were pollinated by Sundrench, and these fruit were less red than fully self-pollinated fruit possibly because the most-common pollen parent, Sundrench, has fruit that are a typically lighter-red than Red Rhapsody fruit.

Insect pollination has been linked to improved strawberry fruit quality [8, 14, 16], but it is unclear whether the fruit size differences between fruit resulting from autogamous pollination and bee- or open-pollination in these studies were caused by more pollen being deposited or more cross-pollen being deposited. The number of fertilised seeds, rather than the pollen source, determines fruit size and mass in Redlands Joy strawberry [15]. In the current study, fruit length, diameter and mass also increased with the number of fertilised seeds and, in Sundrench fruit, with the percentage of fertilised seeds. Partial cross-pollination decreased Sundrench fruit mass. The results of our study suggest that open-pollinated strawberry fruit were larger because more pollen (of any genotype) was deposited on the stigmas. Our results confirm that the number of fertilised seeds determines fruit size [8, 15, 17], making the amount of pollen deposition highly important in this self-compatible crop. Introducing managed bee hives during strawberry flowering or attracting wild pollinators by, for example, establishing high-nature-value pollinator habitat has the potential to increase strawberry yield and fruit quality.

## Conclusion

This study, to our knowledge, is the first to analyse paternity of seeds in an aggregate fruit. We analysed the paternity of nearly 3000 seeds, and identified that strawberry seed filling was almost entirely the result of self-pollination, even at 1 m from a cross-pollen source. Our results confirm that fruit length, diameter and mass depend largely on the number of fertilised seeds. We conclude that high levels of pollen deposition are important for high-quality strawberry production. Introducing bee hives onto farms during flowering and establishing nearby pollinator habitat have the potential to increase strawberry yield and fruit quality.
